# Opinions on the Future of Clinical Pig Kidney Xenotransplantation

**DOI:** 10.3389/ti.2024.13475

**Published:** 2024-11-26

**Authors:** Georg A. Böhmig, Matthias Diebold, Klemens Budde

**Affiliations:** ^1^ Division of Nephrology and Dialysis, Department of Medicine III, Medical University of Vienna, Vienna, Austria; ^2^ Clinic for Transplantation Immunology and Nephrology, University Hospital Basel, University of Basel, Basel, Switzerland; ^3^ Department of Nephrology, Charité Universitätsmedizin Berlin, Berlin, Germany

**Keywords:** clinical transplantation, decedent model, gene-edited pigs, rejection, trial design

## Abstract

Based on promising results obtained in primate models, pioneers in the US have now started to explore the new frontier of genetically-edited pig-to-human transplantation. The recent transition of xenotransplantation into clinical medicine has included transplants in brain-dead subjects and the compassionate use of xenotransplants in living recipients without options for allotransplantation. While the barrier of hyperacute rejection seems to be successfully overcome by gene editing of donor pigs, the occurrence of accelerated rejection could pose significant limitations to the success of the procedure. Ultimately, the establishment of efficient and safe strategies to overcome immunologic barriers will, among other critical factors, such as potential xenozoonotic disease transmission or physiological differences, determine whether and for which indications xenotransplantation will be viable. Considering preliminary outcomes of compassionate use xenotransplantions, which may raise questions about how faithfully data from non-human primate models translate into human outcomes, further research in decedents may be necessary before proceeding with additional clinical transplants. Looking ahead, designing systematic trials in xenotransplantation, including the definition of acceptable eligibility criteria for such high-risk transplants, will be an immense challenge, especially in kidney transplantation, where dialysis provides an effective alternative to transplantation in most cases.

While the authors of this article have no experience in xenotransplantation, neither in experimental research nor in clinical pig-to-human transplants, they have a longstanding scientific interest in strategies for the prevention and treatment of antibody-mediated rejection (AMR) in the context of allotransplantation. Consequently, immunologic aspects—highly relevant also in allotransplantation—dominate their considerations. They are aware that even if the immense immunologic xenobarriers can be successfully managed, other significant factors, such as associated infectious risks, differences in physiology, and important ethical considerations, could come to the forefront [1–3]. Nevertheless, despite these profound challenges, there are sufficient arguments to maximize research efforts towards alternatives to allotransplantation, which, given the speed of development, may include pig-to-human xenotransplantation. Organ transplantation faces an immense organ shortage, posing a considerable burden on the disadvantaged patient group with end-stage organ failure. The transplantation of genetically modified pig organs into humans could potentially fill this gap, avoiding unacceptable waiting times and reducing death rates on waiting lists.

Thanks to major advances in genetic engineering and the establishment of efficient immunosuppressive strategies, recent studies have achieved long-term, rejection-free renal xenograft survival in non-human primates (NHP) [4]. This success was achieved even without the use of CD40/CD154 costimulatory blockade, a strategy shown to reduce immunological risks in xenotransplantation, but not yet approved by the Food and Drug Administration (FDA) for human use [5]. Beneficial long-term results obtained in primate models have finally led pioneers to explore the new frontier of genetically-edited pig-to-human transplantation. We are now witnessing the transition of this concept into clinical medicine, including experimental studies in brain-dead human subjects [6–11], as well as preliminary clinical experiences with pig-to-human xenotransplantation, including heart, kidney, and even liver transplants [12–15].

Actual data on the first human xenotransplants suggest that immunological factors, particularly the occurrence of AMR, are critical in limiting the success of the procedure [16–18]. It could be argued that our ability to establish efficient and safe strategies to overcome immunologic barriers will be decisive in determining whether and for which indications xenotransplantation will be viable. Currently, we do not know how human recipients will respond immunologically to the immense burden of xenoantigen mismatches in the intermediate term, let alone over a long period. Even in short-term experiments, the multifaceted interplay between different components of innate and specific immunity in this context remains poorly defined.

Several centers have now studied porcine xenografts in decedents to explore aspects of immunology, coagulation, infectious disease, and metabolic functions. Using different variants of genetically modified pig kidneys, it has been demonstrated that pig xenotransplants can maintain renal function and provide physiological balance for days up to several weeks [6–11]. A major breakthrough from all these experiments was the effective prevention of hyperacute rejection through genetic modification of donor pigs. Remarkably, while several xenotransplants were performed using pigs with a considerable number of genetic edits, this was achieved also with thymokidneys from pigs with only the alpha-1, 3-galactosyltransferase antigen knocked out [7]. Although the prevention of hyperacute rejection represents a significant step towards successful xenotransplantation, detailed analyses of renal xenotransplants in deceased subjects revealed molecular and morphological features of AMR just a few days after transplantation [17]. In some cases, possibly triggered by a decedent systemic inflammatory process, the occurrence of microvascular injury in the form of thrombotic microangiopathy was observed [6], with recent data supporting utilization of complement inhibition at C5 to control the innate human immune response to porcine kidney xenografts [19].

As part of compassionate use, xenotransplants have been performed in patients with end-stage organ failure. Following two clinical heart xenotransplants in 2022 and 2023, two subsequent clinical kidney xenotransplants were conducted in critically ill patients without alternative options, according to the literature [12, 14]. The first kidney xenotransplant, performed in Boston in March 2024, utilized a gene-edited pig kidney with 69 genomic edits to address immune and coagulation incompatibilities and inactivate porcine endogenous retroviruses. Immunosuppression included costimulatory blockade and complement inhibition. The recipient, however, died in less than 2 months, possibly due to poor underlying health, but data describing the exact circumstances are not yet publicly available [14]. A second kidney xenotransplant at NYU Langone Medical Center in New York City involved a 54-year-old woman with heart and kidney failure. This procedure included a dual transplant of a left ventricular assist device and a gene-edited thymokidney xenograft in a living recipient. The kidney was explanted after several months, but detailed results have not yet been published in a peer-reviewed journal. The latest results from compassionate use clinical transplants—where all recipients (two heart and two kidney) have either died or lost their transplants—raise questions about how accurately data from NHP models translate into human outcomes, where we aim for long-term organ replacement. The authors of this article share the opinion that these results underscore the need for further research before proceeding with additional clinical experiments in living transplant recipients [11].

But how to move forward? After more than 30 years of research with non-human primates, the obvious limitations of this approach are extensively discussed [11]. Likewise, the use of brain-dead decedents raises ethical concerns with a high emotional burden on relatives and staff. But also costs and medical issues due to brain death with all its potential confounding factors restrict the use of experiments involving decedents to short periods. The longest experiment with a decedent was terminated after 2 months and included the reversal of xenograft rejection using multimodal treatment including complement blocking agents [11]. While this case may indicate some incremental achievement, it also demonstrates the difficulties to overcome the strong immunological barriers just for a few weeks. The freedom from AMR months after transplantation in a small cohort of decedents could represent a significant milestone before advancing to living recipients. However, such a requirement will be difficult to achieve in a decedent model due to ethical concerns, costs and long observation time.

Preliminary results indicating a role of early AMR are critically important, particularly given the lack of approved effective treatments to counteract antibody-triggered graft injury even in clinical allotransplantation, where, chronic rejection, often preceded by acute AMR, has emerged as a major cause of accelerated organ loss [20]. We do not know whether new treatments showing promise in allotransplantation will also succeed in xenotransplantation, given the significant genetic disparity between recipient and donor and qualitative differences in rejection processes. However, the xenotransplantation field could benefit from the increased understanding of rejection pathophysiology, particularly the recognized major role of NK cells, which has led to new developments in anti-rejection treatments [21]. For instance, a recent phase 2 trial using a human antibody targeting CD38 showed effective reversal of donor-specific antibody-associated microvascular inflammation, likely achieved through selective depletion of NK cells expressing Fc gamma receptor IIIA [22]. The next steps will be to evaluate whether such innovative treatments could also counteract xenotransplant rejection. It could be argued that the absence of recipient HLA on pig cells may trigger a robust NK cell “missing self” response, which could potentially be addressed by targeting CD38. The role of NK cells in rejection of allo- or xenotransplants, which may involve antibody-independent effector mechanisms, could be significant. However, despite existing therapeutic concepts, the precise pathways underlying DSA-independent injury have yet to be fully defined. This is especially important in xenotransplantation, where various effectors may contribute to intermediate- and long-term xenograft damage. Given that the two primary challenges in transplantation are access to transplants and chronic AMR—the latter a major limitation to long-term allograft survival [23]— it is crucial that these aspects be integrated into xenotransplant research with both urgency and importance.

A key question will be how to progress xenotransplantation to systematic clinical trials beyond compassionate use in isolated patients, particularly considering that initial transplants have not been as successful as anticipated based on primate studies. In the US, individual transplant cases have been conducted with FDA permission through the Expanded Access pathway, including two cardiac and two kidney xenotransplants [24]. For this pathway, three conditions must be met: the patient has a life-threatening illness; there is no therapeutic alternative; and the benefit-risk ratio is favorable. While it is surprising that these authorizations were issued without a clear understanding of the safety and efficacy, this strategy may be useful to gather more data to support future trials [24]. Detailed information on some xenotransplants are not yet available, but this information is crucial for the continuation of clinical transplants. Therefore, transparency and prompt publication of details on failed cases are indispensable prerequisites for progress. However, successful transplantation in patients who are too sick to be listed on a regular waitlist will be difficult. Given the current results, designing trials in xenotransplantation presents immense challenges and multidisciplinary collaborative efforts are needed to overcome these hurdles and develop an ethical path forward towards first studies [25]. The patients who willingly accept the risks associated with participating in xenotransplant trials, which could potentially include accelerated death, will be true heroes. However, how much risk can we ethically allow participants to bear in a trial, and what should the appropriate eligibility criteria be? For heart transplants, it may be relatively straightforward to define eligible cohorts, such as patients with terminal heart failure who are unsuitable for mechanical devices and have a very limited life expectancy. For such patients without a real alternative, even a few months of additional life expectancy may create a benefit. Additional considerations for patient with terminal heart failure are the option of a xenograft as potential bridge to a suitable human heart.

However, the situation is significantly more complex for kidney transplants, as dialysis serves as an effective replacement therapy in the majority of cases, and we would expect a transplant ideally to function for decades and not just a few months. The balance between transplantation and the life-saving but often suboptimal option of long-term dialysis remains a critical issue. It is well established that—depending on donor characteristics, recipient age and the type and severity of underlying medical conditions—the decision to maintain a patient on dialysis versus proceeding with a transplant has a significant impact on patient survival [26]. Any bridging strategy (e.g., xenograft until availability of a suitable human allograft) has also to consider the risks of heavy immunosuppression. A recent European consensus paper suggested including high-risk hemodialysis patients with poor predicted survival or those experiencing difficulties with dialysis access [27]. However, even for these patients, allografts may be available through high urgency listing with 80% patient and 70% graft survival at 5 years [28]. Another consideration could involve including extremely sensitized patients who have no realistic chance of receiving an allograft. Nevertheless, specific allocation programs, like the acceptable mismatch program, along with new therapeutic options such as imlifidase and targeting CD38, could potentially pave the way for successful allotransplantation in such cases [29, 30]. However, even innovative treatments come with inherent limitations. For example, when discussing imlifidase, it is important to note that despite its high efficiency in transient antibody depletion, a major limitation is the rapid reconstitution of HLA antibodies, which restricts its utility in desensitization and treatment protocols [31, 32]. Additionally, it is challenging to predict whether and to what extent a previous xenotransplantation would increase rejection risks in highly alloimmunized patients. A recent model using xenokidneys expressing seven different human transgenes in highly allosensitized rhesus macaques, combined with anti-CD154 monoclonal antibody-based immunosuppression, suggested that prolonged graft survival might be achievable in this high-risk population without the risk of hyperacute rejection triggered by alloantibodies [33]. In this model, allosensitization via serial skin transplantations only temporarily elevated xenoantibodies, and the authors did not observe an increase in alloantibodies post-xenotransplantation or xenograft rejection [33]. Such data are encouraging. However, careful interpretation is warranted, as we have learned that primate data may not necessarily translate to the unique challenges of pig-to-human transplantation.

The clinical trial design in xenotransplantation may not follow conventional rules, and it may be prudent to significantly limit the sample size, especially in the initial phase. For instance, a strategy of sentinel groups, comprising a few treated patients at the outset of the trial before expanding to more participants, may be appropriate. Initial trials must be informed by comprehensive data from preliminary (pre)clinical experiments. This includes establishing the immunosuppressive regimen, covering both induction therapy and maintenance immunosuppression. Additionally, a clearly defined arsenal of rejection treatments is essential, along with diagnostic procedures to guide treatment. This may involve gene expression analyses and the use of biomarkers, such as donor-derived cell-free DNA. The sample size and trial design will require thorough discussion, potentially necessitating an uncontrolled trial setting, including comparisons with matched control groups on dialysis.

Ultimately, what is the primary endpoint and the goal of future clinical trials? As an intermediate goal, a lifesaving bridging strategy may be a reasonable approach to explore the feasibility of a xenograft for a limited period, with the option of a long-term life-sustaining human allograft. The outcomes of such initial trials will determine whether xenotransplantation holds promise as a bridging therapy and could be gradually extended. Their initial results will also critically impact the acceptance of xenotransplantation by patients and the public. Ideally, collaboration among various groups experienced in experimental xenotransplantation, working closely with transplant clinicians involved in highly sensitized transplant programs, and supported by innovative companies advancing gene editing, will be crucial for conducting systematic trials. Let us hope that such collaborative efforts successfully navigate the challenges ahead!

Medical pioneering often encounters setbacks, as exemplified in the initial phase of clinical allotransplantation, where kidney transplantations performed in the 1950s were mostly discouraging and seriously questioned clinical applicability [34]. Nevertheless, a decade later, owing to technical advances and developments in immunosuppression, allotransplantation became a clinical standard of care. The history of transplant medicine underscores how difficult it is to foresee medical triumphs based on initial cases, especially given that progress in medical research typically does not follow a predictable linear path [35]. Undoubtedly, xenotransplantation has made considerable progress over the last 30 years moving from hyperacute rejection minutes after transplant to potentially treatable xenograft rejection after weeks, but there is still a long way to achieve transplant success for decades. We want to conclude with a remark from Sir Roy Calne who, in 1995, said that xenotransplantation “is just around the corner, but it may be a very long corner” [36].

## Data Availability

The original contributions presented in the study are included in the article/supplementary material, further inquiries can be directed to the corresponding author.
